# A randomised controlled trial investigating motor skill training as a function of attentional focus in old age

**DOI:** 10.1186/1471-2318-9-15

**Published:** 2009-05-08

**Authors:** Eling D de Bruin, Jaap Swanenburg, Elsbeth Betschon, Kurt Murer

**Affiliations:** 1 D-Biology, Institute of Human Movement Sciences and Sport, ETH Zurich, Zurich, Switzerland; 2 Department of Rheumatology and Institute of Physical Medicine, University Hospital Zurich, Zurich, Switzerland

## Abstract

**Background:**

Motor learning research has had little impact on clinical applications and rarely extended to research about how older adults learn motor skills. There is consistent evidence that motor skill performance and learning can be enhanced by giving learners instructions that direct their attention. The aim of this study was to test whether elderly individuals that receive an external focus instruction during training of dynamic balance skills would learn in a different manner compared to individuals that received an internal focus instruction.

**Methods:**

This randomised trial included 26 older persons (81 ± 6 years) that were training functional balance twice a week for the duration of 5 weeks. Learning outcomes were recorded after every training session. Weight shifting score and dynamic balance parameters (Biodex Balance System), components of the Extended Timed-Get-Up-and-Go test, five chair rises, and falls efficacy (FES-I) was assessed at baseline and post-intervention.

**Results:**

Participation for training sessions was 94%. No differences between groups were found following 5 weeks of training for weight shifting score, dynamic balance index and dynamic balance time (*p *< 0.95, *p *= 0.16, *p *< 0.50), implying no learning differences between training groups. Extended Timed-Get-Up-and-Go components *Sit-to-stand*, *p *= .036; *Gait initiation*, *p *= .039; *Slow down, stop, turnaround, and sit down*, *p *= 0.011 and the Fes-I (*p *= 0.014) showed improvements for the total group, indicating that function improved compared to baseline.

**Conclusion:**

A 5-week balance training improved weight shifting scores and dynamic balance parameters as well as functional abilities. The observed improvements were independent from the type of attentional focus instructions. The findings provide support for the proposition of different motor learning principles in older adults compared to younger adults.

**Trial Registration:**

ISRCTN44627088

## Background

Motor skills, defined as goal-directed activities that consist of body, head, and/or limb movements [1], are learned and performed on a lifelong basis. Learning, defined as a change in the capability of a person to perform a skill that must be inferred from a relatively permanent improvement in performance as a result of practice or experience, can be assessed by recording practice performance during the period of time a person practices a skill. Skill learning takes place when improvement of the skill over the time period is exhibited [1]. Learning and relearning skills continues to be important for maximising function and quality of life in older individuals. Older adults may, for example, need to train balance skills that will help to reduce the fall risk. One potential way to improve balance training in older adults comes from motor learning research.

Motor learning research has, however, had little impact on clinical applications [2,3], and motor learning research is rarely extended to research about how older adults learn motor skills [4] (also see Latash & Levin [5] for an overview). Older adults, however, constitute a heterogenous group of individuals exhibiting an infinite variety of cognitive and physical abilities. This variety causes learning abilities of the aged to be on a multidimensional continuum ranging from individuals with good learning abilities and good memory skills to individuals with poor learning capacities and impaired memory skills [6].

The learning of motor skills can be characterised by the continuous interaction of cognitive and sensory processes with the motor processes [7]. Decreased postural stability with increasing age can result from impairment in sensory, motor and central integrative mechanisms [8,9]. Teasdale et al. showed that as the sensory information decreased, the postural tasks became increasingly difficult for the elderly and required more of their attentional capacity [10]. Shumway-Cook & Woollacott (2000) reported that the inability to allocate sufficient attention to postural control under multitask conditions may be a contributing factor to imbalance and falls in some older adults [11]. From this point of view, attention resources should explicitly be considered in training protocols for the aged.

There is consistent evidence that motor skill performance and learning can be enhanced by giving learners instructions that direct their attention to the effects of their movements. It has been shown that, given external focus instructions, motor performance can be enhanced above and beyond younger adults' normal level of performance. That is, inducing an external focus of attention (EF) has been shown to be more effective than directing attention to the movements themselves (internal focus; IF) or to some other external cue that will prevent learners from focussing on their movements [12]. Thus, the performer's focus of attention has an important influence on the performance and learning of motor skills [12-15].

Postural biofeedback balance training for the improvement of dynamic stability can be applied by weight shifting to selected targets displayed on a computer screen [16]. Such visual feedback-based balance training improves the dynamic balance and sensory integration capabilities of older adults with a history of falls [17]. It is, furthermore, able to improve balance control in the physically active community-dwelling elderly [18] and in frail elderly women living in residential care [19]. It also decreases the monthly risk of falling in frail elderly [20].

Uncertainty exists about the impact of subtly different instructions from the screen cursor and the effect on movement quality. Rose & Clark (2000) [17] report that "the visual feedback conveys information to the participant about the movement and position of his/her centre of gravity during each exercise". Assuming that, on a behavioral level, age differences in motor skill learning are at most only subtle [4], this form of information should be extended with an 'external focus instruction' in order to facilitate the motor learning process of the older performer.

This study applied two different types of attentional focus instructions to a group of frail older adults training dynamic balance skills while measures of learning progress were obtained. We tested the hypothesis that elderly individuals that receive an external focus instruction during training of dynamic balance skills would show different patterns of learning compared to individuals that received an internal focus instruction.

## Methods

### Participants

Thirty-one participants were recruited from two senior citizens' hostels and their surroundings in Zurich, Switzerland for a randomised controlled, single-blind trial. Recruitment was performed by means of word-of-mouth recommendation and poster advertisement. Inclusion criteria were 70 years of age and older, the ability to see the feedback marker on the computer screen, a score of 25 or more in the Mini Mental Status Examination (MMSE) [21], the ability to be able to follow verbal instructions in the German language and the ability to stand upright independently. Participants were excluded if they had a rapidly progressive or terminal illness, acute illness or unstable chronic illness (self-reports that were cross checked with the physician's reports). Participants who were undergoing balance training at the time of enrolment or had prior experience with the task were also excluded.

All eligible residents were invited to attend an information session in which the intervention and the study design were explained. Participants were informed that the purpose of the study was to investigate rehabilitation strategies. The Ethical Committees of the Canton Zurich and the Swiss Federal Institute of Technology Zurich approved the study protocol and informed consent was obtained from all participants prior to their participation.

### Randomisation

**Figure 1:**
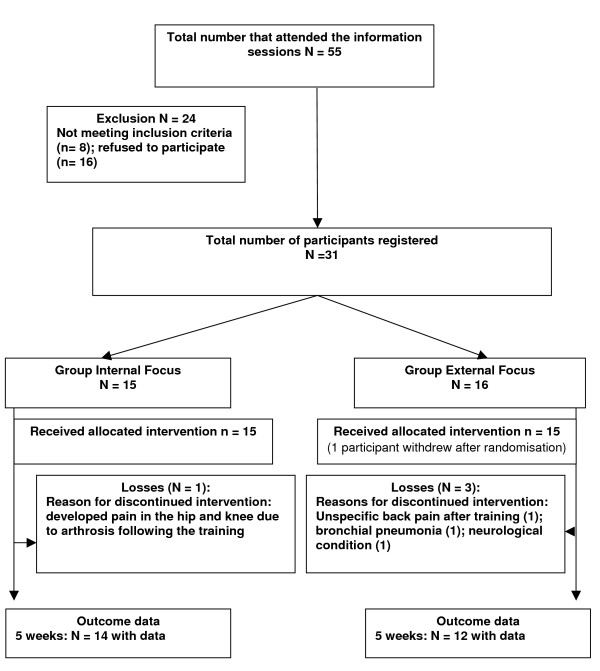
**Study design – flow of participants through the study**.

Participants were allocated randomly to the internal focus of attention (IF) group or the external focus of attention (EF) group. Participants were randomised using a table of random numbers [22]. Figure 1 shows the recruitment process and the flow of participants through the study.

### Device and task training

Balance assessment and training were carried out using the Biodex Stability System (BSS; Biodex Medical Systems Inc, 20 Ramsay Rd, Shirley, NY 11967–4704). The BSS trains and evaluates neuromuscular control by quantifying the ability to maintain dynamic postural stability on both stable and unstable surface conditions. The BSS provides visual feedback that allows individuals to relate to and reproduce specified motion patterns. All participants trained in non-slip socks when possible.

**Figure 2:**
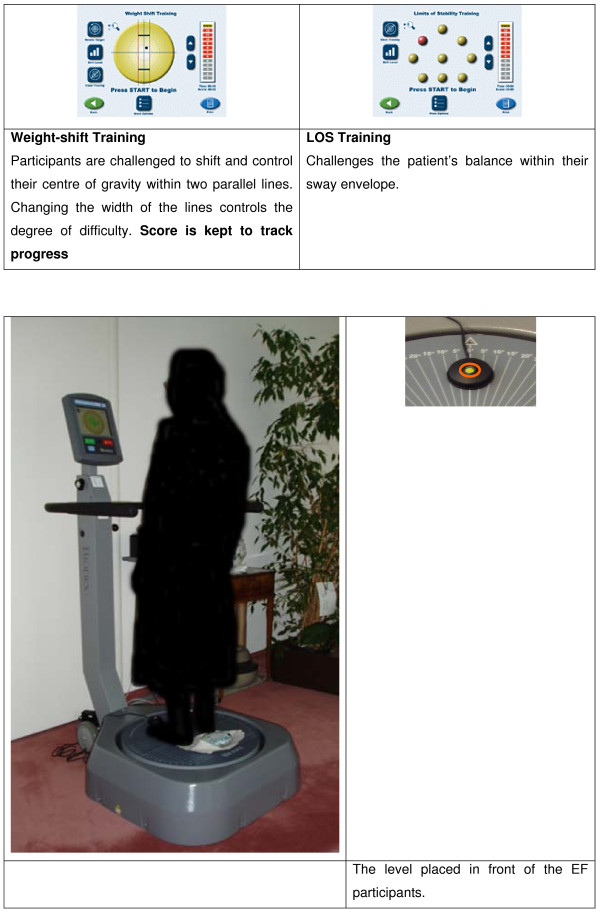
**a Weight-shift training modality (left), LOS training and test modality (right)**. **b **Exercise set-up with the water blister placed in front of the feet of EF participants.

Participants in both groups were instructed to focus on the visual feedback screen whilst performing their exercises. The screen showed a moving point in the middle of a target and the participants were asked to follow the target through shifting their weight. All participants received an exercise protocol in which the exercises gradually became more complex (Additional file 1; Figure 2).

The **IF **group participants were, prior to internal focus trials, instructed with the feedback that the moving point on the feedback screen represented their body centre of gravity. They were instructed to focus on this centre through concentrating on their belly (while looking straight ahead at the video screen) and to volitionally move this point on the screen through exertion of force on this imaginary point.

The **EF **group participants were instructed that the moving point on the feedback screen represented an air bubble in a level, (as used in the building industry to determine a level horizontal line), positioned in front of the feet of the participant standing on the plate (Figure 2). Such a level (Ø 4.2-cm) was attached to the platform with an electronic cable, thus suggesting a direct connection between movements of the air bubble in the level and the marker on the screen (figure 2). The EF participants had to focus on the air bubble (while looking straight ahead at the video screen) and try to consciously move this bubble in the level.

All participants were expected to perform at least three practice trials per exercise on two week days for five consecutive weeks under the treatment conditions. Before every practice trial, participants were given short reminders to focus on the respective 'moving points'. In a fourth trial, the time needed to perform the exercise under test conditions was measured together with the Dynamic Stability Index of the participants. This procedure was chosen to rule out short-term learning effects [23,24]. Training duration lasted between 25 and 35 minutes.

Participants were informed that the intent of the trial was to assess the effect of balance training on physical performance. In order to avoid bias, the information that different focus of attention instructions during training possibly influences the speed of learning was not disclosed. Complete information about the theoretical role that the form of instruction might play was given to each participant in a debriefing session after completion of the programme. The reason for not disclosing information about the focus of attention was discussed at the debriefing. Only the results of participants who agreed with this procedure were included in the final data analysis.

### Primary outcome variables

The weight shift score for medio-lateral movements on the stable platform, performance time on the unstable platform for the dynamic limits of stability (LOS) test and the dynamic stability index derived from this test were measured in each session and were used as the primary outcomes. The device measures angular displacement from a center position. The stability index units are really degrees. In the static mode – it is the angular displacement of the center of gravity. In the dynamic mode, it is the angular displacement of the platform that is measured with accelerometers that measure tilt with respect to gravity. Test conditions were similar to exercise conditions (Figure 2a) with the exception that during the test a time pressure and/or precision constraint was imposed on the participants.

The weight shift training mode allows for static and dynamic platform exercise in the most basic activities of weight shifting. Participants were challenged to shift and control their centre of gravity during the weight shift test within two parallel lines for sixty seconds. During the test the width of the lines was kept constant and, thus, the degree of difficulty was kept constant for the 5-week study time. Participants were asked to move the dot on the feedback screen from left to right between the blue lines without touching the red boundary lines with the dot (see figure 2a). Scoring is percentage based and equals total targets accumulated/total hits within 60 seconds. During the test participants were not allowed to hold the security rails (Figure 2a).

In the LOS test condition, participants were asked to volitionally move their body within their LOS on an unstable platform (level 10 representing medium difficulty) in as short a time as possible. Time to complete the test (in seconds) is the total elapsed time for the participant to complete touching all targets. This test is a good indicator of dynamic control within a normalised sway envelope [25]. The LOS has been defined as the maximum angle a body can achieve from vertical without losing one's balance. Once the LOS is exceeded a fall, stumble or step will ensue. LOS in normal adults is 12 degrees in anterior-posterior (AP), and 16 degrees in the medio-lateral (ML) direction. For the BSS, the percentage of normal LOS in the AP direction is calculated by dividing the subject's AP stability index (maximum 20°) by a normal reference value of 12° and multiplying that number by 100. Similarly, subject's ML stability index (maximum 20°) is divided by a normal reference value of 16° and multiplied by 100 to determine the percentage of normal LOS in the lateral direction [26]. A combined value of 100% means perfect control. Values below indicate problems with dynamic balance. Reliability for a dynamic balance protocol with decreasing stability levels on the BSS has been proven clinically reliable, with intraclass correlation coefficients ranging from .80 to .43 [27]. A combined value of 100% for the stability index meant perfect control. Values below indicated problems with dynamic balance. The time needed to perform this test was documented in seconds.

### Functional performance variables

The Timed Get-up-and-Go (TGUG) test measures the overall time to complete a series of functionally important tasks. In the Expanded Timed Get-up-and-Go (ETGUG) test, times for the component tasks are measured using a multi-memory stopwatch. The ETGUG test is a sensitive and objective assessment of function [28].

The time for 5 consecutive chair rises without the use of hands was recorded. Hands were folded in front of the chest with feet flat on the floor, following the protocol described by Guralnik et al. [29] Time was measured in seconds with a stopwatch and rounded to the nearest tenth of a second. Timing began with the command "Go" and ended when the buttocks contacted the chair on the fifth landing. The reliability of this protocol is adequate [29].

The Falls Efficacy Scale International (FES-I) questionnaire was used as a measure of 'concern' about falling to determine the transfer effects of training. This scale assesses both easy and difficult physical activities and social activities with a scale of: 1 = not at all concerned, 2 = somewhat concerned, 3 = fairly concerned, 4 = very concerned. The FES-I has excellent internal and test-retest reliability [30].

All measurements were performed by the same tester. The secondary variables were assessed at baseline and after completion of 10 trainings. The assessor was not blinded to the participants' training group.

### Data analysis

Baseline group data that were parametric and showed a normal distribution were evaluated using Student's *t*-test for unpaired groups. A comparison of group score and data not normally distributed at baseline was undertaken using a Mann-Whitney *U *test. The Chi-squared test was used for dichotomous variables.

The primary outcome data were analysed with 2 (Focus groups) × 10 (Days) ANOVAs, with repeated measures on the second factor. Missing data were replaced with a conservative approach that would not influence the mean of the whole distribution, by means that were calculated from available data prior to analysis [31]. Omega squared (ω^2^) was calculated to determine the amount of variation that was accounted for by the difference in the two levels of attentional focus instruction (IF & EF) [32]. Variables in which ANOVA assumptions were violated (e.g. normality of the distribution) were compared by two-way ANOVA by ranks using Puri and Sen's *L*-statistic [33], where Pillai's Trace as an estimate of variance accounted for in repeated measures is used instead of ω^2^.

The functional performance outcome data ETGUG and Chair Rises were analysed with two-factor repeated-measures ANOVA, with external/internal focus of attention as a between-participants factor and time as a within-participants factor. For statistical comparisons of the before-after FES-I data we used the Mann-Whitney *U *test. A probability level of p < 0.05 was considered to be statistically significant.

All statistical procedures were conducted with the SPSS (version 15.0) software program (SPSS Inc. Chicago, IL, USA). All available data were analysed by initial group assignment.

## Results

The progress of participants through the various stages of the study and the resulting compliance for both groups is presented in Figure 1. All participants agreed with the information procedure at the end of training and had their data included in the final data analysis.

### Participant description

Table 1 summarises the demographic characteristics and baseline measures of the two groups at the beginning of the trial. There were no significant group differences for these measures.

**Table 1:** Participants' demographics and baseline measures

	Internal focus (n = 14)	External focus (n = 12)	*p*
Age (years), mean ± SD (n = 26), [range]	80.1 ± 5.4 [72–92]	81.9 ± 6.8 [71–98]	0.46^†^

Gender F/M (n = 26)	10/4	11/1	0.33^†††^

Height (m), mean ± SD (n = 26)	1.62 ± 0.07	1.60 ± 0.07	0.50^†^

Mass (kg), mean ± SD (n = 26)	67.9 ± 9	65.5 ± 16.5	0.27^†^

Medication (number/day), mean ± SD	6.5 ± 4.3	4.6 ± 3.3	0.30^†^

Mini Mental Status Examination, mean ± SD	28.5 ± 0.6	28.2 ± 0.7	0.08^†^

FES-I, mean ± SD	21.8 ± 4.6	23.9 ± 7.8	0.78^††^

5 chair rises test (s), mean ± SD	16.1 ± 2.9	18.1 ± 4.4	0.28^††^

ETGUG (s), mean ± SD	29 ± 13.2	37.7 ± 22	0.22^††^

^†^*t*-test; ^††^Mann-Whitney *U*-test; ^†††^Chi-squared test.

### Compliance

From the 26 participants that completed the trial 50% visited all 10 scheduled training sessions, 10 participants visited 9 sessions, and three completed 8 training sessions. This resulted in a total compliance, expressed as *trainings visited (244) *divided by *trainings scheduled (260) *× 100% = 94%, indicating good programme acceptance. There were no differences in compliance between the two groups nor were there group differences in the rates of progression through the training stages.

### Primary outcomes

All 26 participants were able to perform the exercises and the subsequent weight-shift test on the stable platform and the exercises on the unstable platform. However, five participants were not able to perform the exercises under the imposed time pressure and precision constraints that followed every training session. These individuals missed four or more test values which led to their exclusion from data analysis. Complete data sets with seven or more test values for the performance time and dynamic stability test measure was available for 21 individuals; 12 in the IF and 9 in the EF group.

ANOVA assumptions for normality of the distribution were violated for the weight-shift score and the time needed for the dynamic test.

**Figure 3:**
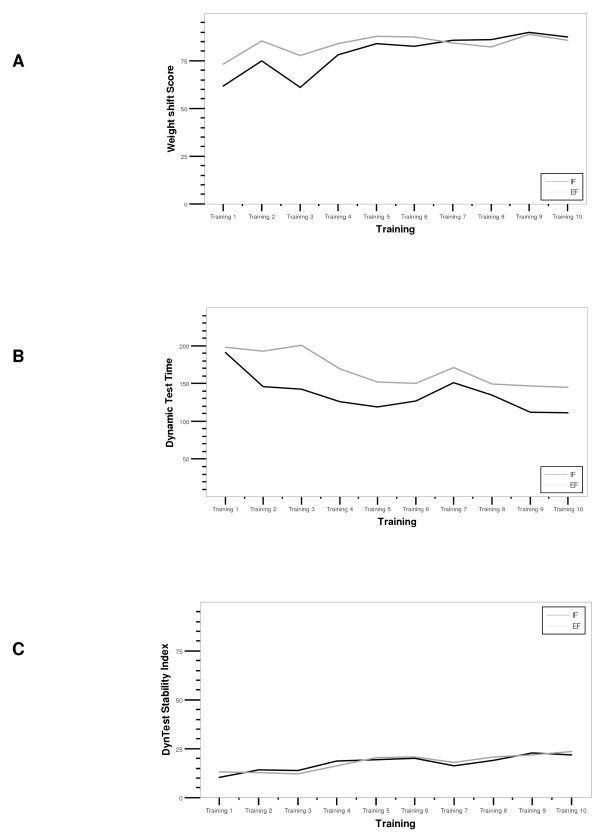
**Biodex Balance System performances of the internal and external focus groups with visual feedback during practice (week 1–5)**. Curves represent the development of the three primary outcome variables weight shift score (A), dynamic LOS performance time (B), and dynamic LOS index (C).

The between groups tests indicated that the variable external/internal focus of instruction failed to reach significance in weight shift; *L*(20) = 3.28, *p *< 0.95, and dynamic test performance time; *L*(20) = 7.06, *p *< 0.50. The within subject tests indicated that there was a significant time effect for both parameters: *L*(20) = 16.58, *p *< 0.05 and *L*(20) = 15.86, *p *< 0.05. In other words, both groups similarly improved in task performance within 5 weeks. Pillai's Trace was .13 for weight shift and .39 for performance time (Figure 3). This means that a thirteen (weight shift) respectively 39% (dynamic test time) proportion of the variance in dynamic stability is accounted for by the external/internal focus of instruction variable.

The between groups test indicated that the variable external/internal focus of instruction failed to reach significance in dynamic stability index *F*(1, 19) = 2.133, *p *= 0.161, ω^2 ^= .05. The within subject test indicated that there was a significant time effect for this parameter: *p*-value <0.001. In other words, both groups similarly improved in task performance within 5 weeks. A five percent proportion of the variance in dynamic stability is accounted for by the external/internal focus of instruction variable (Figure 3).

### Functional performance variables

The two groups did not vary significantly with respect to ETGUG, Chair Rises and FES-I. Neither were there significant differences in post-test values between the two groups. The lack of any significant differences related to focus of instruction led to the dissolution of the grouping and all participants were treated thereafter as a single group and analysed with a *t*-Test for paired observations (ETGUG & Chair Rises) or the Wilcoxon signed-rank test (FES-I).

**Figure 4:**
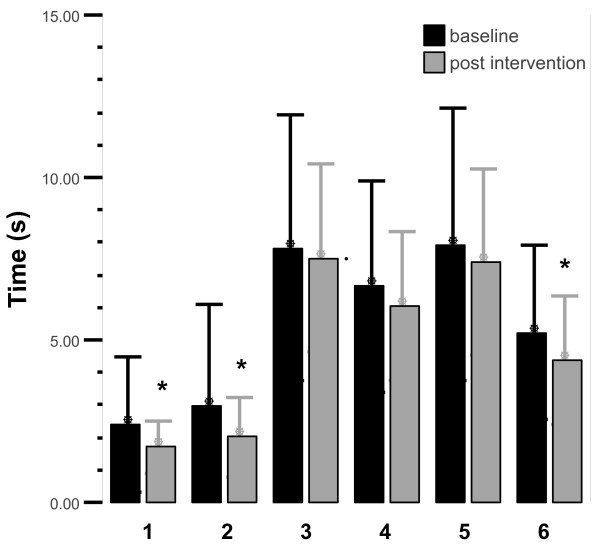
**The component tasks of the ETGUG test; 1 = *Sit-to-Stand*; 2 = *Gait initiation; *3 = Walk 1; 4 = *Turn around*; 5 = Walk 2; 6 = *Slow down, stop, turn around, and sit down***. * Signifies a significant change following training.

The analysis for the ETGUG total time score before (33.0 ± 18.0s) and after training (29.1 ± 11.4s) showed that there was no change in this parameter, *t*(25) = 2,01, *p *= .056. Three of the six component tasks, however, showed significant improvements over the 5-week training time; *Sit-to-stand*, *p *= .036; *Gait initiation*, *p *= .039; *Walk *1, *p *= .89; *Turn around, p *= .33; *Walk 2*, * p *= .44; and *Slow down, stop, turnaround, and sit down*, *p *= 0.011 (Figure 4).

Five of the participants were not able to perform the Chair Rises test at baseline. At follow up four of these participants were not able to perform this test. Thus the analysis was performed with 21 participants. The analysis for the Chair Rises showed that there was no change in this parameter when before training (17.1 ± 3.7s) was compared to follow up (16.4 ± 3.5s), *t*(20) = 1,38, *p *= .182.

The Wilcoxon test on the FES-I data showed that there was significant difference in fall efficacy between pre-training (22.8 ± 6.2 points) and post-training (20.8 ± 3.6 points), *z *= -2.468, *p *= 0.014, indicating that the concern about falling decreased.

## Discussion

This study tested the hypothesis that elderly individuals using different attentional focus strategies conveyed to the participants through differing verbal instructions during training of dynamic balance skills would show differences in learning progress. Although there is consistent evidence in the motor learning literature that motor skill performance and learning can be enhanced by giving learners instructions that direct their attention to the effects of their movements, our study showed that there was no such effect on learning in older adults. Neither the primary test variables nor the functional performance parameters showed that external focus instruction was beneficiary to internal focus instruction whilst learning to balance on a stable or unstable platform with concurrent feedback on a video screen.

How motor processes are affected by internal versus external foci is explained through the constrained action hypothesis. According to this view, focussing attention on the movement effect promotes an automatic mode of movement control [13,34,35]. It is assumed that through the adoption of an external focus unconscious, fast and reflexive processes are enabled to control the movements. Evidence in support of the constrained action view are related to attentional capacity, the frequency of movement adjustments, and the degree of muscular activity observed under different focus conditions. A possible explanation for the lack of effect in our study might be related to these factors. The implications for learning motor skills from verbal instructions are, in the majority, derived from motor learning experiments with healthy, young adults. We investigated frail older individuals training balance skills, a population that is more representative for many rehabilitation settings. It may be argued that this population may have had impairments that influence the automaticity of movements in either one or all of the aforementioned factors related to the constrained action view. Impairments in older individuals in attention, movement adjustment capacities, and/or muscular activity in standing balance are well documented (see [36] Chapter 9 for an overview).

Our single-activity intervention showed significant changes on components of the ETGUG test measure, independent from the form of verbal instructions. These results are in line with previous studies that used similar methodology [17-19] or investigated the impact of balance and coordination training alone on locomotor function [37]. Hass et al. reported a positive influence of balance and coordination training on gait initiation in older adults that were transitionally frail [37]. Our study is at variance, however, with the results from Steadman et al. (2003) who reported improvements in walking velocity of balance-impaired patients following a balance training programme [38]. Steadman et al., however, specifically included functional exercises that aimed to improve walking and leg strength in their exercise protocol. This finding suggests that the principles of specificity of training may be as important in older adults as they are in younger adults. For example, specificity may explain why our results differ from those of Steadman, et al. (2003). In their study, both sit-to-stand and walking exercises were important components of the balance training programme [38]. Weight-shifting exercises on a stable and unstable platform were the only exercises that were performed in our study. Therefore, the Chair Rise Test, developed to test the strength in the legs of the elderly, showed no change in our training group.

The intervention also indicated that concern about falling decreased as shown through significant changes in the FES-I. These changes were independent from the form of verbal instructions and infer that the intervention was able to influence the level of fear to perform functional indoor and outdoor activities. These results seem to confirm findings in previous studies that used similar methodology and comparable training duration, showing that this form of intervention improves balance control, dynamic balance and sensory integration capabilities of older adults [17-19].

An aspect of this study that should be viewed with some caution is the operationalisation of internal versus external focus of attention. It can well be argued that both task conditions require the same kind of behaviour (shifting one's weight in order to follow the target). Once the people with the external focus of attention instruction realised that the movement of the bubble corresponds directly to their body movements on the platform, they might simply have shifted their weight, just as the people with the internal focus of attention instruction did. This would have led both groups to focus on the movements effects (which are the movements of the feedback point in this paradigm) But, if that would be the case, the results of previous studies with young adults, that were using similar experimental set-ups and similar forms of instructions, would in majority also reveal similar patterns in training effects. Young adults, however, mainly showed different performances depending on internal and external focus of attention.

An obvious limitation of our study is the rather small sample size. This study, therefore, only reveals first estimates for these measures and warrants further research in larger populations. We implemented a strict study design to control for threats to validity. A next step would be to replicate the findings in a new exercise group of elderly individuals as an additional control procedure [39]. The data collected allow for calculation of the sample size needed for a larger study. The ANOVA for the Dynamic Stability Index, for example, showed that the means were not different and the effect size was small with the omega squared being just .05. This indicates that the factor *focus instruction *by itself accounted for only 5% of the overall (effect+error) variance. To avoid a type I or II error in a future study we need, based on our observed values for the Dynamic Stability Index (with values in the last training of: IF = 26.6 ± 12.8; EF = 32.5 ± 14.1), an estimated sample size of 88 participants for a two group pretest-posttest design. This would result in 80% power at an α-level of 0.05 and is based on the assumption that the standard deviation of the response variable is 13.5. This in mind, the relationship between focus of instruction research and its effect on motor learning in elderly individuals requires further exploration. Translating the results from motor learning experiments with healthy young participants to therapeutic interventions should, therefore, take place with caution until the appropriate clinical studies with clinically relevant population outcomes have been conducted.

Further research is needed to determine how older individuals training functional motor skills respond to different types of focus instructions and transfer this to these motor skills. Current knowledge could serve as a good starting point.

## Conclusion

In summary, we found a similar degree in the learning rate between two groups of older adults exercising a postural balance task under different instructions. Our findings provide support for the proposition of different motor learning principles in older adults compared to younger adults, because the older individuals were not taking an advantage of adopting an external focus of attention.

## Competing interests

The authors declare that they have no non-financial competing interests (political, personal, religious, academic, ideological, intellectual, commercial or any other) to declare in relation to this manuscript.

## Authors' contributions

EdB conceived of and designed the study, performed the statistical analysis and wrote the manuscript. EB carried out the training intervention and helped to draft the manuscript. JS participated in its design. KM helped to draft the manuscript and revised the manuscript critically for its content. All authors read and approved the final manuscript.

## Pre-publication history

The pre-publication history for this paper can be accessed here:



## Supplementary Material

Additional File 1 **Description of training activities and five-week progression schedule**. The data provided describe the training activities performed and include the five-week progression schedule. Click here for file
