# Supplementary material for: A randomised controlled trial investigating motor skill training as a function of attentional focus in old age
Source: BMC Geriatr. 2009 May 8;9:15. doi: 10.1186/1471-2318-9-15 (PMC2684534; doi:10.1186/1471-2318-9-15)
Supplement: Additional File 1 — Description of training activities and five-week progression schedule. The data provided describe the training activities performed and include the five-week progression schedule. [file 1471-2318-9-15-S1.pdf]

Week	Task	Surface	Imposed constraint
1	Single goal task: weight-shifting in seated or standing position within broad parallel lines (Note: all participants were able to perform the task in standing position):<br>Task self paced with 100% visual feedback.<br>Weight shifting modes<br>• Anterior-posterior (1 Min)<br>• Medio-lateral (1 Min)<br>• Left & right diagonal (1 Min)<br>LOS training (5 Min)<br>LOS Test	Broad standing position; Stable surface for weight shifting exercise; unstable surface for LOS training & test.	No external constraints imposed
2	Single goal task: weight-shifting in seated or standing position within broad parallel lines:<br>Task self paced with 100% visual feedback.<br>Weight shifting modes	Broad standing position; Stable surface for weight shifting exercise; unstable surface for LOS training & test.	During training of weight shifting tasks the parallel lines will be smaller (this in contrast to the test condition where the degree of difficulty was kept constant for the 5-week study time)

	• Anterior-posterior (1 Min)<br>• Medio-lateral (1 Min)<br>• Left & right diagonal (1 Min)<br>LOS training (5 Min)<br>LOS Test		
3	Single goal task: weight-shifting in standing position within broad parallel lines:<br>Task self paced with 100% visual feedback during test condition.<br>Weight shifting modes<br>• Anterior-posterior (1 Min)<br>• Medio-lateral (1 Min)<br>• Left & right diagonal (1 Min)<br>LOS training (5 Min)<br>LOS Test	Variable standing positions; Stable surface for weight shifting exercise; unstable surface for LOS training & test.	Manipulate stance positions[†] (base of support); ask performer to halve the broad standing position<br>Impose time constraint during weight shifting exercises.
4	Single goal task: weight-shifting in standing position within various parallel lines:<br>Task self paced with 100% visual	Unstable surface conditions	Manipulate stance positions (base of support); ask performer to either halve the broad standing position or to take a semi-tandem position

	feedback during test condition.<br>Weight shifting modes<br>• Anterior-posterior (1 Min)<br>• Medio-lateral (1 Min)<br>• Left & right diagonal (1 Min)<br>LOS training (5 Min)<br>LOS Test		Impose time constraint during weight shifting exercises.<br>Manipulate sensory information (standing on foam during training).
5	Single goal task: weight-shifting in standing position within broad parallel lines:<br>Task self paced with 100% visual feedback during test condition.<br>Weight shifting modes<br>• Anterior-posterior (1 Min)<br>• Medio-lateral (1 Min)<br>• Left & right diagonal (1 Min)<br>LOS training (5 Min)<br>LOS Test	Unstable surface conditions	Manipulate stance positions (base of support); ask performer to either halve the broad standing position or to take a semi-tandem position.<br>Impose time constraint during weight shifting exercises.<br>Manipulate sensory information (standing on foam during training).

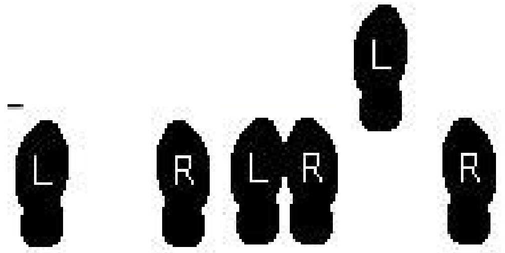

† Manipulation of the stance position. Subjects started with a hip wide foot standing position and progressively narrow their base of support through side-by-side standing(middle) and/or semi-tandem standing position (right). All subjects were able to progress to all stages.
